# Supplementary material for: Rhizoferrin Glycosylation in Rhizopus microsporus
Source: J Fungi (Basel). 2020 Jun 18;6(2):89. doi: 10.3390/jof6020089 (PMC7344610; doi:10.3390/jof6020089)
Supplement: Supplementary file 1 [file jof-06-00089-s001.pdf]

Supplementary Material

Rhizoferrin glycosylation in *Rhizopus microsporus*

Anton Škríba [1], Rutuja Hiraji Patil [1,2], Petr Hubáček [3], Radim Dobiáš [4], Andrea Palyzová [1], Helena Marešová [1], Tomáš Pluháček [1,2], and Vladimír Havlíček [1,2,*]

[1] Institute of Microbiology of the Czech Academy of Sciences, Vídeňská 1083, 142 20 Prague, Czech Republic;
[2] Dept. of Analytical Chemistry, Faculty of Science, Palacký University, Olomouc, Czech Republic;
[3] Dept. of Medical Microbiology, Charles University and Motol University Hospital, Prague, Czech Republic; [4] Public Health Institute in Ostrava, Ostrava, Czech Republic
* Correspondence: vlhavlic@biomed.cas.cz

Contents:

1. Standard addition
2. Exact tandem mass spectrometry

1. Standard addition

For determination of the RHF and bis-imido-RHF contents in fermentation broths, 3 aliquots of resuspended samples (45 µL, 3-fold diluted solution) were transferred to new vials, and then, mixed RHF and bis-imido-RHF standard solution or 5% ACN (nonspiked sample) was added to obtain a final volume of 50 µL. Since the final concentration of analytes changed for each point of the standard addition curve, the added volumes of mixed bis-imido-RHF and RHF standard and 5% ACN varied for each aliquot (for example, in the first fortified sample, 5 µL of the mixed bis-imido-RHF and RHF standard was added). Then, prepared samples were subjected to UHPLC-HRMS analysis in triplicate. Finally, a standard addition curve was constructed by plotting the peak area at each point against the added RHF and bis-imido-RHF concentration used for the spikes. The analyte contents in fermentation broths were determined as the value of the intercept of the curve on the x-axis. The concentration of imido-RHF presented in the main article was calculated using data for bis-imido-RHF (we assume an equal response factor) as the corresponding imido-RHF standard is not available on the market.

Fresh fermentation broth sample:

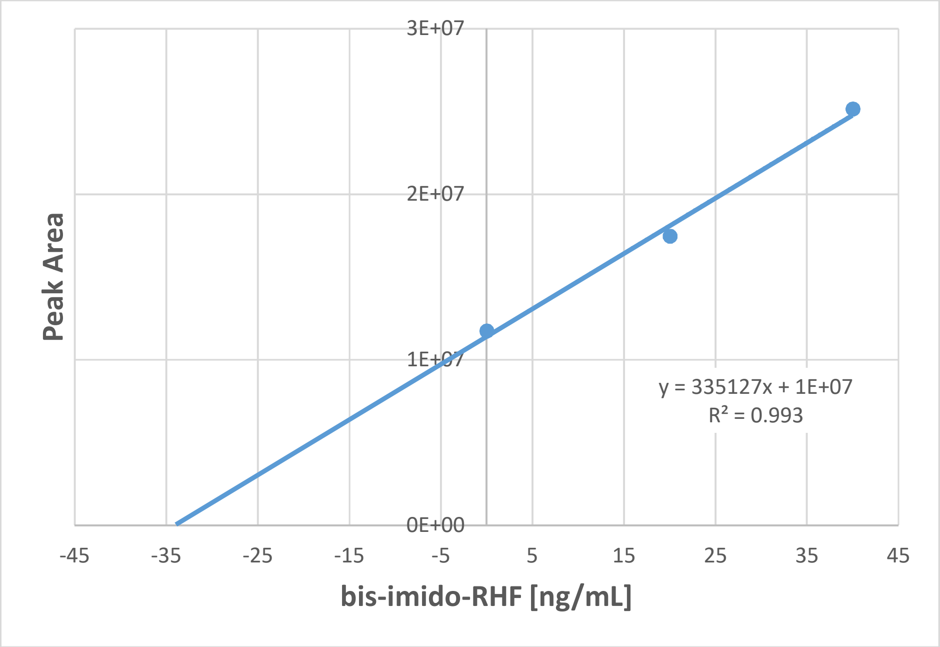

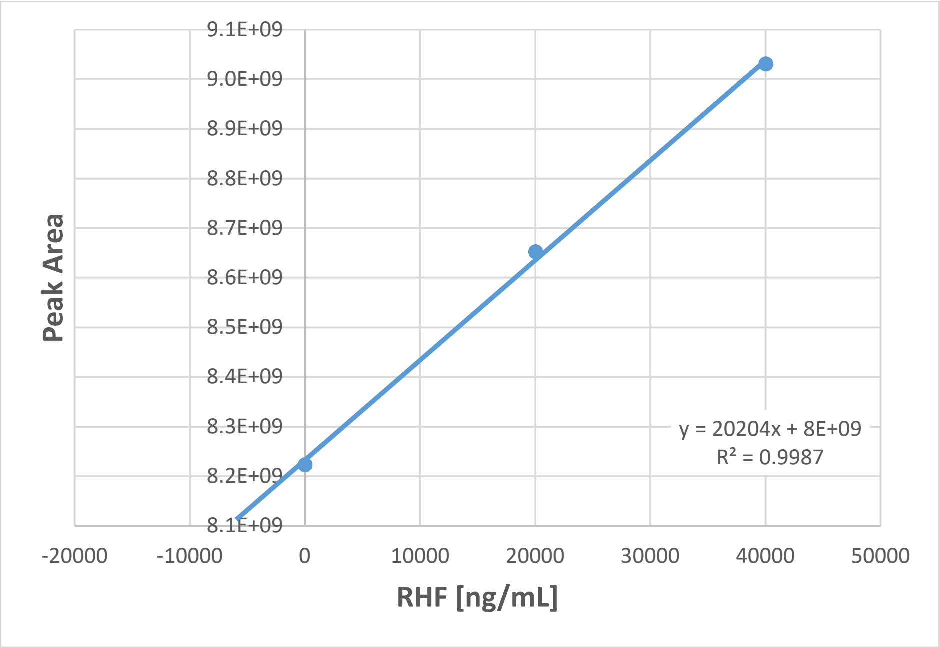

Five-month-old fermentation broth sample:

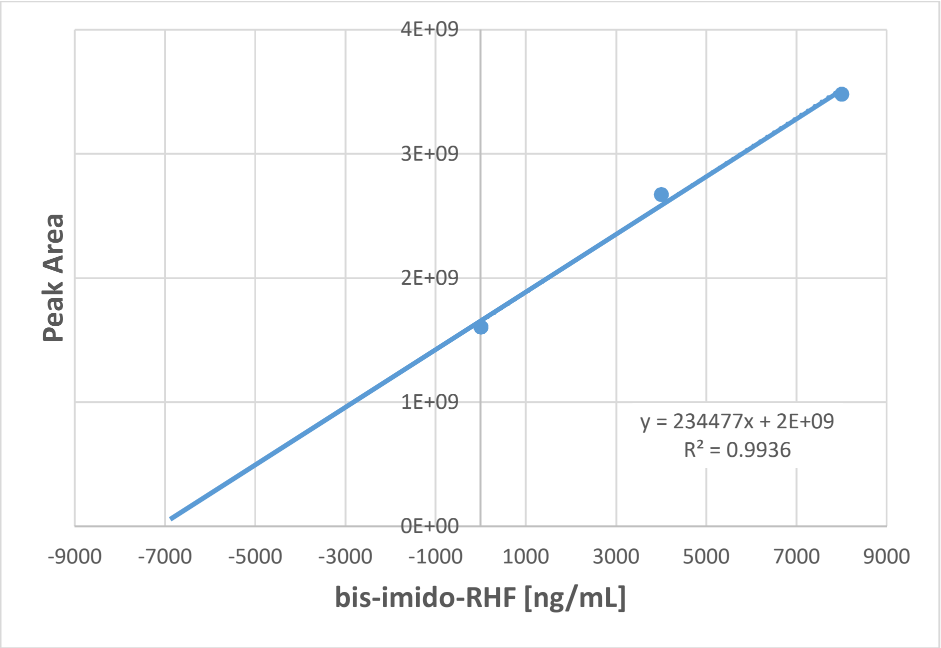

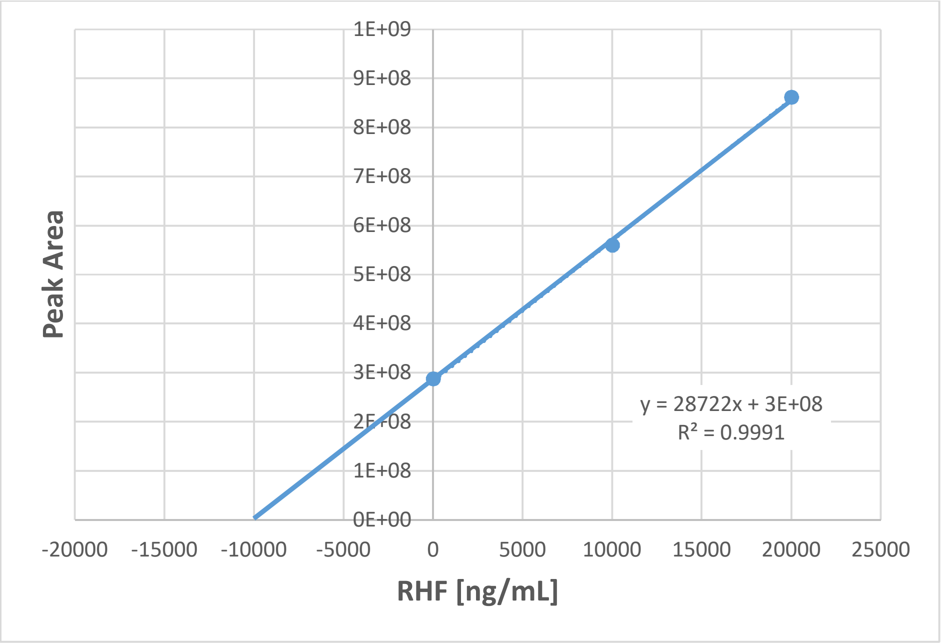

2. **Exact tandem mass spectrometry**

m/z measured		m/z		
Parent	Fragment	Calculated	Composition	Error (ppm)
1085.34769	1085.34769	1085.35149	$C_{40}H_{65}N_2O_{32}$	3.50
	1067.34629	1067.34093	$C_{40}H_{63}N_2O_{31}$	-5.02
	1049.33566	1049.33036	$C_{40}H_{61}N_2O_{30}$	-5.04
	1031.32399	1031.31980	$C_{40}H_{59}N_2O_{29}$	-4.06
	905.29277	905.28811	$C_{34}H_{53}N_2O_{26}$	-5.15
	887.28193	887.27754	$C_{34}H_{51}N_2O_{25}$	-4.20
	869.27122	869.26698	$C_{34}H_{49}N_2O_{24}$	-3.25
761.24601	761.24601	761.24585	$C_{28}H_{45}N_2O_{22}$	-0.21
	743.23536	743.23528	$C_{28}H_{43}N_2O_{21}$	-0.20
	725.22485	725.22471	$C_{28}H_{41}N_2O_{20}$	-0.19
	707.21462	707.21415	$C_{28}H_{39}N_2O_{19}$	-0.74
	689.20344	689.20358	$C_{28}H_{37}N_2O_{18}$	0.20
	599.19375	599.19302	$C_{22}H_{35}N_2O_{17}$	-1.21
	581.18313	581.18245	$C_{22}H_{33}N_2O_{16}$	-1.17
	563.17241	563.17189	$C_{22}H_{31}N_2O_{15}$	-0.92
	545.16200	545.16133	$C_{22}H_{29}N_2O_{14}$	-1.22
	527.15150	527.15076	$C_{22}H_{27}N_2O_{13}$	-1.40
437.14016	437.14016	437.14020	$C_{16}H_{25}N_2O_{12}$	0.09
	419.12947	419.12963	$C_{16}H_{23}N_2O_{11}$	0.38
	391.13475	391.13472	$C_{15}H_{23}N_2O_{10}$	-0.07
	373.12415	373.12415	$C_{15}H_{21}N_2O_9$	0.00
	355.11356	355.11359	$C_{15}H_{19}N_2O_8$	0.08
	337.10294	337.10303	$C_{15}H_{17}N_2O_7$	0.26
	329.13442	329.13433	$C_{14}H_{21}N_2O_7$	-0.28
	319.09255	319.09246	$C_{15}H_{15}N_2O_6$	-0.29
	263.12390	263.12376	$C_{10}H_{19}N_2O_6$	-0.53

	245.11339	245.11319	$C_{10}H_{17}N_2O_5$	-0.81
	228.08675	228.08665	$C_{10}H_{14}NO_5$	-0.431241
